# Dynamic microfluidic control of supramolecular peptide self-assembly

**DOI:** 10.1038/ncomms13190

**Published:** 2016-10-25

**Authors:** Zohar A. Arnon, Andreas Vitalis, Aviad Levin, Thomas C. T. Michaels, Amedeo Caflisch, Tuomas P. J. Knowles, Lihi Adler-Abramovich, Ehud Gazit

**Affiliations:** 1 Department of Molecular Microbiology and Biotechnology, George S. Wise Faculty of Life Sciences, Tel Aviv University, Tel Aviv 69978, Israel; 2 Department of Biochemistry, University of Zurich, Winterthurerstrasse 190, CH-8057 Zurich, Switzerland; 3 Department of Chemistry, University of Cambridge, Lensfield Road, Cambridge CB2 1EW, UK; 4 Department of Oral Biology, The Goldschleger School of Dental Medicine, Sackler Faculty of Medicine, Tel Aviv University, Tel Aviv 69978, Israel; 5 Department of Materials Science and Engineering, Iby and Aladar Fleischman Faculty of Engineering, Tel Aviv University, Tel Aviv 69978, Israel

## Abstract

The dynamic nature of supramolecular polymers has a key role in their organization. Yet, the manipulation of their dimensions and polarity remains a challenge. Here, the minimalistic diphenylalanine building block was applied to demonstrate control of nano-assemblies growth and shrinkage using microfluidics. To fine-tune differential local environments, peptide nanotubes were confined by micron-scale pillars and subjected to monomer flows of various saturation levels to control assembly and disassembly. The small-volume device allows the rapid adjustment of conditions within the system. A simplified kinetic model was applied to calculate parameters of the growth mechanism. Direct real-time microscopy analysis revealed that different peptide derivatives show unidirectional or bidirectional axial dimension variation. Atomistic simulations show that unidirectional growth is dictated by the differences in the axial ends, as observed in the crystalline order of symmetry. This work lays foundations for the rational control of nano-materials dimensions for applications in biomedicine and material science.

## Abstract (web summary)

The organization of supramolecular peptide polymers determines their properties; however, controlling their dimensions still remains a problem. Here, Gazit *et al*. show the spontaneous elongation and shortening of these polymers at an individual nano-assembly level by using a microfluidic platform.

Supramolecular polymers have been extensively studied due to their notable chemical and physical properties, ease of production and functional diversity^1^,^2^,^3^,^4^,^5^.The dynamic nature of supramolecular polymers is the basis for attributes such as self-healing^6^, structural modulation^7^ and controlled reorganization^8^,^9^,^10^. These qualities are highly desirable for nanotechnological device development for future applications in biomedicine and materials science^11^,^12^,^13^. Yet, the control over growth manner and size is still an unmet challenge.

In contrast to covalent polymers, non-covalent interactions impart on supramolecular polymers the advantage of dynamic architectural transitions and modularity. The non-covalent interactions between protein building blocks are also important in self-organizing cellular systems, such as microtubules^14^. The dynamic characteristics of the microtubules allow the reversible nature of the intracellular filament system. The establishment of applicable and reproducible supramolecular polymer models, as well as direct visualization and manipulation of the assembly kinetics and dynamics, are of key importance for the mechanistic understanding and utilization of the involved structural transitions on the molecular level of biological and bio-inspired systems.

The diphenylalanine (FF) building block self-assembles into nanotubes in a spontaneous manner through a well-ordered and consistent process^15^,^16^,^17^. FF assemblies exhibit unique physical properties including robust mechanical rigidity, ferro- and piezo-electricity, luminescence and semiconductivity^18^. Various FF derivatives have been shown to self-assemble into a range of different morphological end-products with diverse physical properties^19^,^20^,^21^,^22^. Control of the assembly dimensions as well as the morphology of the formed structures has, to date, mainly been induced by varying the organic solvents and peptide concentrations used^22^,^23^.

Here, the FF nanotubes were subjected to various conditions in order to demonstrate control of nano-assemblies axial association and dissociation using a microfluidic platform. Since the study of a single FF nanotube in aqueous solution is limited in bulk, we chose to meet this challenge by compartmentalization of individual tubes and visualization of the assembly process. Preformed peptide nanotubes were restricted by micron-scale pillars within a microfluidic device and exposed to monomer flows of various saturation levels to manipulate the assembly process. By adjusting the building block concentration in the small-volume device, we were able to rapidly control the net monomer influx at given time points. Due to the design of the device, the flow within the channels was limited to a laminar one and thus the distribution of building blocks could be considered homogenous. Direct real-time microscopy analysis revealed that different peptide derivatives show either unidirectional or bidirectional axial dimension variation. *K*_on_ and *K*_off_ analysis provided the basis for a kinetic model of the growth mechanism and these parameters were calculated. Molecular dynamics simulations show that unidirectional growth is due to differences in the axial ends, as observed in the crystalline order of symmetry. This microfluidic platform can provide insights into self-assembly mechanisms and kinetics that could be utilized for the design and fabrication of dynamically controlled nano-devices useful in the fields of bionanotechnology and nano-materials.

## Results

### Controlling the assembly process of FF nanotubes

**Figure 1:**
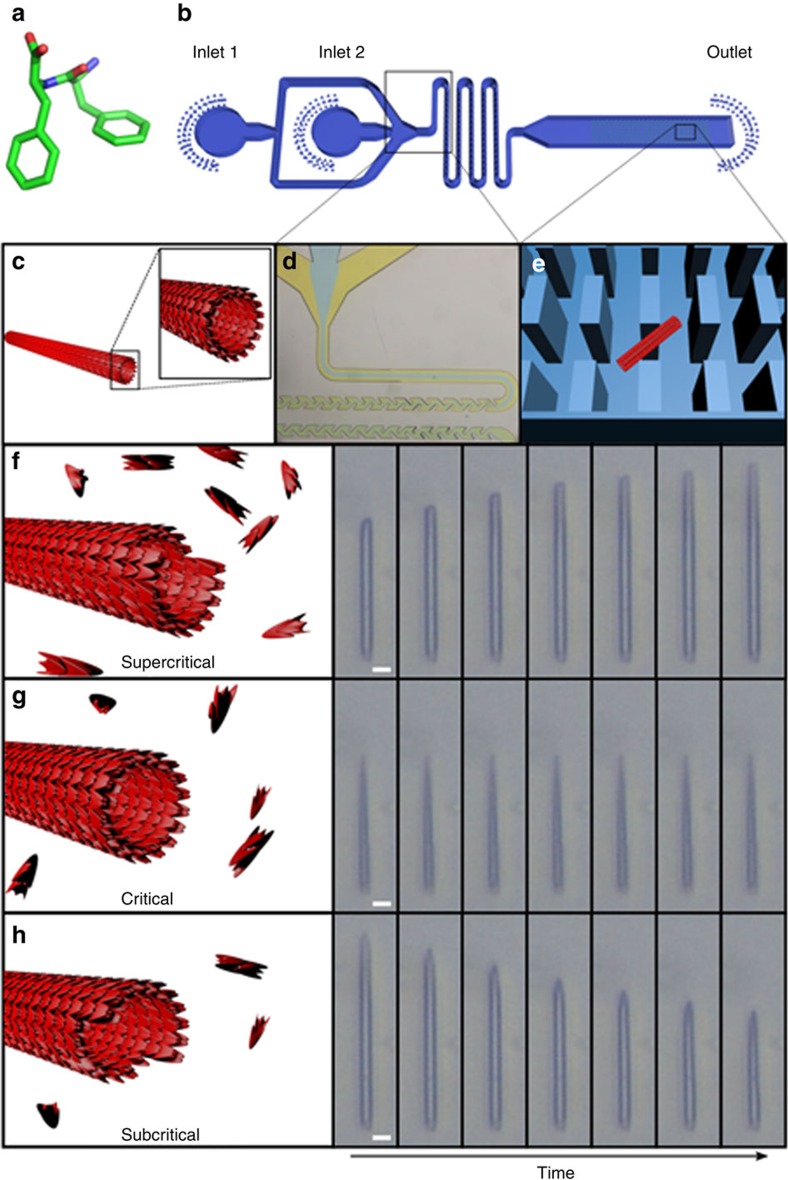
Dependency of unidirectional FF nanotube elongation and shortening on free monomer concentration. (**a**) Crystalline conformation of an FF monomer. (**b**) Architecture of the microfluidic device. Two inlets, followed by a long and narrow mixing channel and a wider channel with micro-scale pillars. (**c**) Nanotube illustration. (**d**) The narrow mixing channel ensures the two solutions are mixed properly, as demonstrated with yellow and blue food colouring solutions. (**e**) Illustration of a nanotube confined by the micro-scale pillars. (**f**–**h**) Illustration and imaging of FF nanotubes. A series of seven images at different time points under supercritical (3.20 mM) (100 s interval between images) (**f**) critical (2.43 mM) (60 s interval between images) (**g**) and subcritical (1.60 mM) (10 s interval between images) (**h**) concentrations. Supercritical concentration resulted in the elongation of the nanotubes. At the critical concentration, the assembly and disassembly rates are equal. The length of the same nanotube at a subcritical concentration decreases due to the reduced assembly rate. Scale bars, 5 μm.

Initially, preformed FF nanotubes were injected into a microfluidic device and a portion of these structures were confined by micron-scale poly(dimethylsiloxane) (PDMS) pillars (Fig. 1b,e, Supplementary Movie 1 and Supplementary Data 1). The pillars were designed to allow the confinement of the structures, as well as avoid obstruction of the microfluidics channel (see microfluidic device and microfluidic experiments sections of Supplementary Methods for further information). While most nanotubes were not confined by the pillars, a portion of the structures was restricted and could not drift with newly injected solutions. As a result of the device's small volume, the solution surrounding the nanotubes could be adjusted within seconds by injecting the desired solvent and peptide concentration. Fully dissolved peptide solutions of subcritical and supercritical building block concentrations were introduced into the microfluidic device. Control over building block concentrations at the solution phase at a range of 0.5–1 mg ml^−1^ (1.60–3.20 mM) was then achieved by injecting subcritical and supercritical FF solutions at different ratios of flow rates into the device; the elongation or shortening behaviour of the structures was examined using light microscopy and was evidently dependent on the building block concentration.

In order to ensure proper mixing of the two solutions within the channel, yellow and blue food colouring solutions were introduced into inlets 1 and 2 of the microfluidics device (Fig. 1d and Supplementary Fig. 1). At the starting point of the mixing channel there was a clear phase separation between the yellow and blue solutions. After approximately one-tenth (1.5 mm) of the mixing channel length, the two colours were indistinguishable and a green solution was apparent. Injecting the solutions in either high or low flow rates gave similar mixed green solution long before approaching the wide channel of the device in which the crystals were immobilized.

The length of the tubes was measured at different time points in order to determine the elongation dynamics. As the critical concentration of FF in water is 0.76 mg ml^−1^ (2.43 mM) (ref. 22), a solution of 1 mg ml^−1^ (3.20 mM) FF was used as the supercritical concentration. While the width of the nanotubes did not change noticeably, the length of the tubes increased over time (Fig. 1f). Under quiescent conditions, building blocks incorporated into the nanotubes and free building blocks in solution reach an equilibrium state. Conversely, in the microfluidic device, the nanotubes are subjected to a continuous flow of monomers, and equilibrium is never reached. In order to achieve the critical concentration of 2.43 mM, solutions of 3.20 and 1.60 mM were injected to the microfluidic flow reactor at rates of 2.2 and 1.7 μl h^−1^, respectively. Under these conditions, the assemblies remained consistent in length throughout the experiment, confirming the assumption that the calculated critical concentration is reached (Fig. 1g). In a subcritical environment (1.60 mM), building blocks composing the nanotubes had disassociated and the structures decreased in length (Fig. 1h). Due to the constant flow of the solution, the dissociation of building blocks from the nanotube does not increase the free monomer concentration of the solution; hence, the structures continue to shorten until their complete degradation. Interestingly, the entire set of analysed FF nanotubes revealed a unidirectional growth and shortening behaviour (Fig. 1f–h). Unlike the aligned growth of FF nanotubes on solid surfaces using physical vapour deposition^19^, in which only one termini of the grown structure is accessible, the conditions using the microfluidics settings allow growth on both termini; regardless, the assemblies elongate in a unidirectional manner. To our knowledge, this unidirectionality phenomenon has never been reported, most likely due to the lack of suitable methodologies to visualize confined individual nanotube growth in real-time.

### Controlling the assembly process of CycloFF assemblies

**Figure 2:**
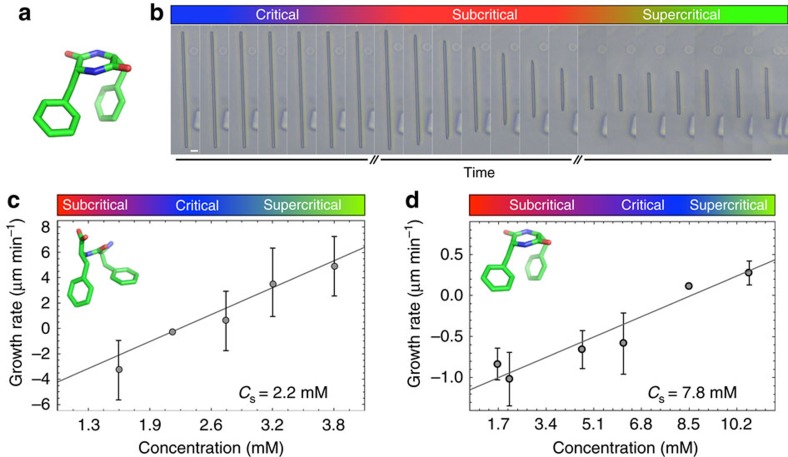
FF and CycloFF nanotubes growth rates. (**a**) Crystalline conformation of a cycloFF monomer. (**b**) Elongation and shortening of preformed cycloFF nanotubes. At its critical concentration, the nanotube showed no change in length (5 min interval between images). Dissociation and elongation of the nanotube were demonstrated at subcritical (4 min interval between images) and supercritical (30 min interval between images) concentrations (1.70 and 13.59 mM), respectively. (**c**) FF nanotube growth rate displays a linear behaviour with increasing peptide concentration. (**d**) CycloFF nanotube growth rate as a function of increasing peptide concentration and best fit to the linear relationship *R=k*_on_* × c−k*_off_. Each data point represents the average growth rates of ten individual nanotube termini. Scale bar, 10 μm. Error bars represent the standard error of the growth rate of 10 individual tubes.

To further explore the possibility of quantitative control over the self-assembly product axial dimensions, we next focused on the supramolecular assemblies formed by the cyclo-(Phe-Phe) (cycloFF) dipeptide. CycloFF is an FF derivative, which also forms needle-like assemblies. The crystallographic arrangement of the cycloFF structures shows an orthorhombic packing, where each dipeptide molecule forms a pair of hydrogen bonds with adjacent molecules^24^. Due to the significant hydrophobicity, we chose to dissolve the cyclic peptide in dimethyl sulfoxide (DMSO) to actualize self-assembly. CycloFF solutions in DMSO at 0.5 mg ml^−1^ (1.70 mM) and 4 mg ml^−1^ (13.59 mM) were used as subcritical and supercritical conditions, respectively. The length of the tubes remained unchanged throughout the experiment when solutions at flow rates of 1.9 and 2.1 μl h^−1^ were injected. The calculated final critical concentration for cycloFF in DMSO was found to be 2.34 mg ml^−1^ (7.95 mM). However, unlike in the case of FF in double distilled water (DDW), the elongation and shortening manner of cycloFF is bidirectional (Fig. 2b). It is important to note that FF in DMSO self-assembles into plate-like structures (Supplementary Fig. 2) that do not display a morphology which constitutes an elongated axis, the radial or axial growth behaviour displays little similarity to that of FF in double distilled water or CycloFF in DMSO, thus, comparison of CycloFF in DMSO and FF in DMSO is unproductive.

### Kinetic model

In order to get insights of the growth and shortening mechanism, kinetic analysis was applied. Nanotube growth occurs through an elongation and dissociation events, where the addition of monomers at growing ends with rate *k*_on_* × c* (μm min^−1^) acts to lengthen the nanotubes, and the loss of subunits at a rate *k*_off_ (μm min^−1^) acts to shorten them. Here *k*_on_ (μm min^−1^ mM^−1^) is the elongation rate and *c* is the local concentration of monomers near the growing end. The net growth rate *R* of FF nanotubes is a linear function of the local monomer concentration *c*, *R=k*_on_* × c−k*_off_, where *k*_on_∼3.36 (μm min^−1^ mM ^−1^) and *k*_off_∼7.4 μm min^−1^ (Fig. 2c). Thus, the rate constants ratio for dissociation and association *c*_s_*=k*_off_ *k*_on_^−1^∼2.2 mM is the critical concentration. The assembly process consists of two consecutive steps: nucleation and maturation. Nucleation is a statistical process dependent on the volume of the solution. Maturation, is the elongation of a preformed aggregate. Our results demonstrate a linear dependency of the growth rate on the peptide concentration in solution. This simplified model is valid only to the linear process of assembly maturation of preformed structures. Similarly, the growth of CycloFF assemblies is characterized by the parameters *k*_on_∼0.15 (μm min^−1^ mM ^−1^) and *k*_off_∼1.2 μm min^−1^, corresponding to a critical concentration of *c*_s_*=*k_off_ k_on_^−1^∼7.8 mM (Fig. 2d). We note that our analysis neglects the potential effect of the uneven mass transfer of subunits to the individual nanotube ends caused by microfluidic flow. To investigate this potential effect, we measured cycloFF growth rates for each elongating terminus separately and we found that the resulting rates could not be distinguished from each other within experimental error (Supplementary Fig. 3), indicating that mass transfer of subunits to nanotube ends is not a determining factor in this case. It is important to state that all tubes, regardless of solution concentrations, eventually halted from elongating, probably due to significant defects in the crystal packing, and subsequently capped.

### Molecular dynamics simulations

**Figure 3:**
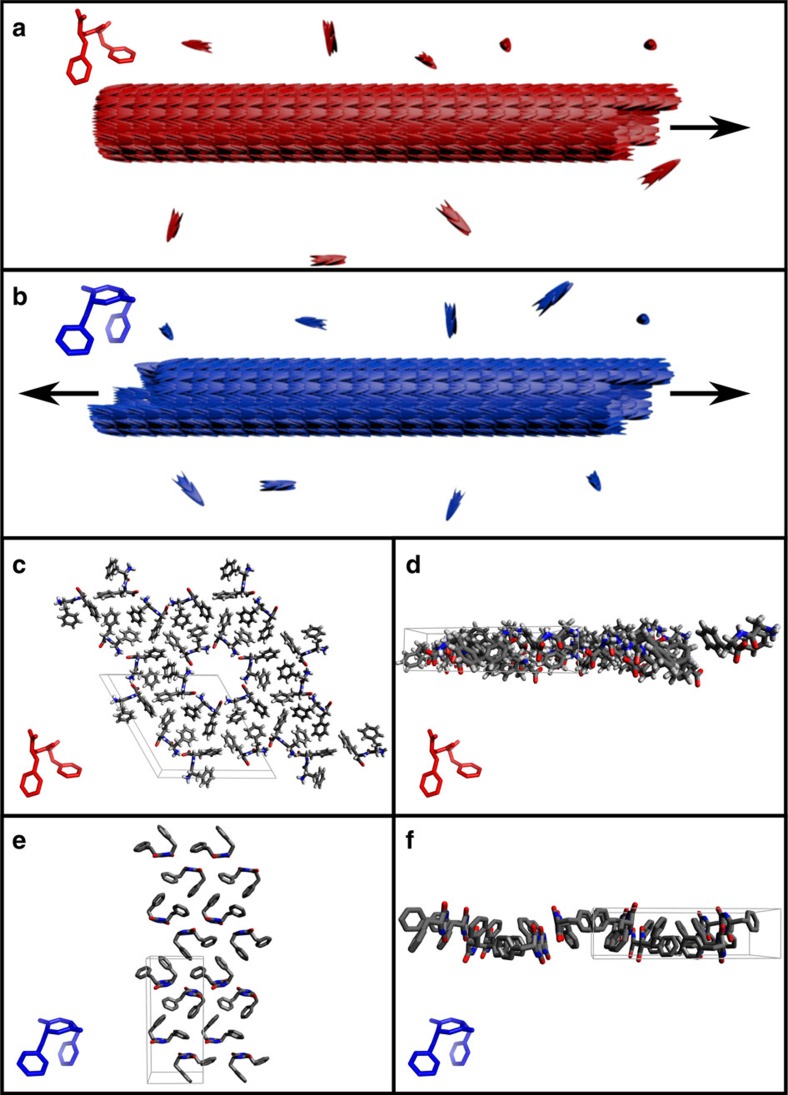
Crystal structure of FF and cycloFF peptides. (**a**,**b**) Illustration of growing FF and cycloFF tubes: unidirectional elongation of FF (**a**) and bidirectional elongation of cycloFF (**b**). (**c**,**d**) Packing of FF crystal structure^25^. The view along the *c* axis shows the symmetry of axes *a* and *b*. (**c**) The asymmetry along the *c* axis becomes apparent when viewed along the *a* axis (**d**). (**e**,**f**) The symmetry of all three axes of cycloFF crystalline structure^24^ is revealed when viewed via the *c* (**e**) and *a* axes (**f**).

In order to interpret the observed unidirectional and bidirectional growth behaviours, we examined the crystal structures of the FF and cycloFF assemblies (Fig. 3c–f). While both the *a* and *b* axes are symmetrical in the FF crystal structure, the *c* axis, which is considered to be the elongation axis of the nanotube^25^, is asymmetric in nature, as the majority of oxygen atoms point in one direction of the *c* axis (Fig. 3d). In contrast, all three axes of the cycloFF crystal are symmetric (Fig. 3e,f). Due to the cycloFF symmetry, new associating building blocks cannot differentiate between the two termini of the nanostructure, which, therefore, undergoes a bidirectional growth. Conversely, the two termini of the *c* axis of FF tubes are distinctly different; this could affect the affinity of one end for the addition of free building blocks in comparison to the other. In order to examine the correlation between the elongation behaviour and the crystalline order of symmetry, we inspected different assemblies with known crystal structures. These assemblies exhibited the same correlation of elongation behaviour with the crystalline order of symmetry. For example, GC dipeptide nucleic acid (di-PNA) showed a bidirectional elongation manner, as the crystal structure of the assembly is symmetrical for all three axes^26^. On the other hand, the growth of 2-(*N*-propinol)-5-nitropyridine is unidirectional^27^, and the nitrogen in the pyridine ring introduce assymetry along one axis of the crystal structure^28^ (data not shown).

**Figure 4:**
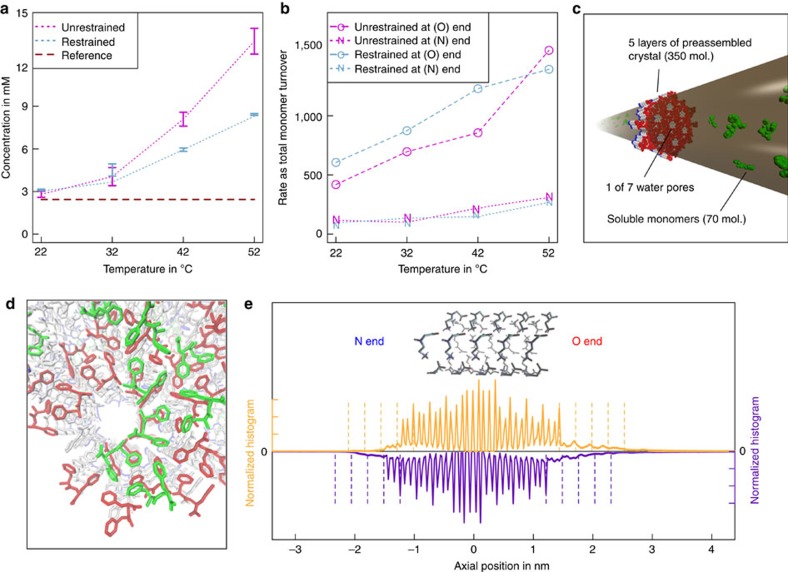
**Molecular** **simulations of FF nanotubes growth.** Two data sets were utilized, one with and one without monomer-level conformational restraints towards the crystal conformer. (**a**) The remaining concentration of soluble monomers at equilibrium as a function of temperature. Error bars indicate the variation between the third and fourth quarters of the total simulation length. The critical concentration is indicated as a reference. (**b**) Rate of monomer turnover at the O (fast) and N (slow) interfaces as a function of temperature. (**c**) Schematic view of an initial configuration for simulations. The periodic cylinder is shown in bronze. The central layer and molecules protruding from the cylinder are frozen throughout. (**d**) Final simulation snapshot of a part of the O interface for the restrained case. Molecules found initially in the soluble phase (green) have adsorbed in-registry to the top layer at the O end (red). (**e**) Axial density profiles of carboxyl (left *y* axes) and amino (right *y* axes) groups for the restrained case. Vertical dashed lines indicate the most prominent peak positions expected for a systematic continuation of the crystal. The schematic crystalline structure depicts the crystal orientation.

To corroborate the observed FF growth asymmetry, we performed molecular simulations of a slice of the crystalline assembly (Fig. 3c,d). By virtue of using an implicit solvent model^29^, we achieved realistic bulk concentrations in a cylinder of length 0.188 μm that is aligned with the asymmetric crystal axis. One-dimensional periodicity ensures that both distinct interfaces can be probed by the same soluble phase. A mixed Monte Carlo^30^ and molecular dynamics^31^ sampler was used. Details on simulation set-up and analysis are provided in the molecular simulations section of the Supplementary Discussion. Since simulations are performed in a constant particle number ensemble and without flow, we used a match of the remaining soluble concentration at equilibrium and the measured critical concentration to demonstrate the appropriateness of the model (Fig. 4a). Across four different simulation temperatures, the best match is with 22 °C, close to room temperature. Importantly, Fig. 4b shows that total binding and unbinding rates both differ strongly at the two interfaces. The net assembly rate is limited by the amount of soluble species and becomes zero at equilibrium. The presence of an interface with reduced rates for both binding and unbinding is consistent with the results in Figs 1 and 2 that exclude a model where both interfaces have comparable rates, but inverted ratios of binding versus unbinding. These conclusions are independent of whether the cost of the conformational search at the monomer level is prepaid by means of dihedral angle restraints or not.

The face of the assembly with high solvent-accessibility of carboxylates rather than amino groups exhibits faster exchange. Indeed, the polarity of quasi-helical assemblies of peptide backbones in the structure is the main source of asymmetry (Fig. 3d). Figure 4d,e demonstrate that this asymmetry propagates in simulations. Ordered growth is possible, if all molecules are internally restrained to crystal-compatible conformers (Fig. 4d). Without these restraints, a more amorphous growing tip forms, yet both types of growth preserve the aforementioned polarity (Fig. 4e and Supplementary Fig. 4).

## Discussion

In conclusion, we have directed the spontaneous elongation and shortening of supramolecular peptide polymers at an individual nano-assembly level through the use of a microfluidic platform, allowing for physical confinement and controlled manipulation of the chemical potential of the building blocks. Variations in the solution concentration (subcritical, critical or supercritical) dictate association or dissociation of the supramolecular polymers. Control over the concentration within the device was achieved in real-time by flow rate variation through the inlets of the microfluidic device. This feature further allowed us to determine the critical concentration of CycloFF in DMSO. Furthermore, the kinetic analysis of the growth rate versus monomer concentration, revealed the mechanism underlying the association–dissociation rates. We could clearly establish either unidirectional or bidirectional dimension alteration for various building blocks. Molecular simulations provided strong evidence that the growth behaviour is restricted by the crystalline order of symmetry facilitated by the specificity of the monomer addition mechanism. These differential patterns observed with symmetric and asymmetric crystals validate our hypothesis. Moreover, the combined microfluidic experiments and atomistic simulations will allow the rational design of supramolecular polymers with desired attributes and behaviour, based on the nature of monomer incorporation into the formed nanostructures. Control over both the dimensional alteration and kinetics as well as the growth directionality can provide a new set of tools for the design of supramolecular polymers for a wide range of future applications in biotechnology and material science.

## Methods

### Materials

Peptides were purchase from Bachem, Switzerland (purity ≥97%). Fresh stock solutions were prepared by dissolving FF in water at concentrations of 3.20 and 1.60 mM and cycloFF in DMSO at a concentration of 1.70 and 13.59 mM. Preformed structures were assembled by dissolving FF in water at concentration of 6.40 mM and cycloFF in DMSO at concentration of 33.97 mM and heating to 90 °C. Structures were visible when samples were cooled down to room temperature.

### Microfluidic device

Microfluidic device was designed using CleWin software (CleWin Layout Editor Version 4.1.2.0, Micro and Nano Fabrication and Characterization Facility, Tel Aviv University) (Supplementary Movie 1) and fabricated with PDMS, using SU8 on silicon masters and standard soft lithography techniques. Inlets and outlets were punched and PDMS was then plasma bonded to glass slides to create sealed devices. The main channel of the device, which includes the pillars, is 1 mm wide and 8 mm long. Pillars dimensions are 50 × 25 μm. The width mixing channel varies berween 30–100 μm. The height of the channel is 50 μm.

### Microfluidic experiments

Preformed crystalline structures were inserted into the device. Then, a flow of solutions of known concentrations was injected at a rate of 4 μl h^−**1**^ using Cetoni GmbH neMESYS Syringe Pumps (Korbussen, Germany) and glass HAMILTON syringes, 1,725 TLL of 250 μl. This process was examined under an OPTIKA XDS-2 Trinocular Inverted microscope, and images were captured at different time points. See microfluidic platform section of Supplementary Discussion for further information.

### Image analysis

Captured images were analysed using ImageJ 1.45S. The length of the tubes was measured at all time points. Growth and shortening rates were calculated by including only those tubes, which had both ends visible in the image frame for the entire process. The length of each end was measured for five times and averaged for each time point.

### Crystal structure

Crystal structures 2 × 2 × 2 packing images were illustrated using Mercury 3.3. Crystal monomer images were represented using PyMol 1.3.

### Molecular simulations

All details regarding the setup and analysis of molecular simulations are provided in the molecular simulations section of the Supplementary Discussion. Images were generated using VMD 1.9.2 and R 2.14.

### Data availability

The authors declare that the data supporting the findings of this study are available within the article and its Supplementary Information files

## Additional information

**How to cite this article:** Arnon, Z. A. *et al*. Dynamic microfluidic control of supramolecular peptide self-assembly. *Nat. Commun.* **7,** 13190 doi: 10.1038/ncomms13190 (2016).

## Supplementary Material

Supplementary Information Supplementary Figures 1-4, Supplementary Discussion, Supplementary Methods and Supplementary References

Supplementary Movie 1 Microfluidics Device Illustration

Supplementary Software 1 Design of the microfluidics device.
