# Supplementary material for: Dynamic microfluidic control of supramolecular peptide self-assembly
Source: Nat Commun. 2016 Oct 25;7:13190. doi: 10.1038/ncomms13190 (PMC5093325; doi:10.1038/ncomms13190)
Supplement: Supplementary Information — Supplementary Figures 1-4, Supplementary Discussion, Supplementary Methods and Supplementary References [file ncomms13190-s1.pdf]

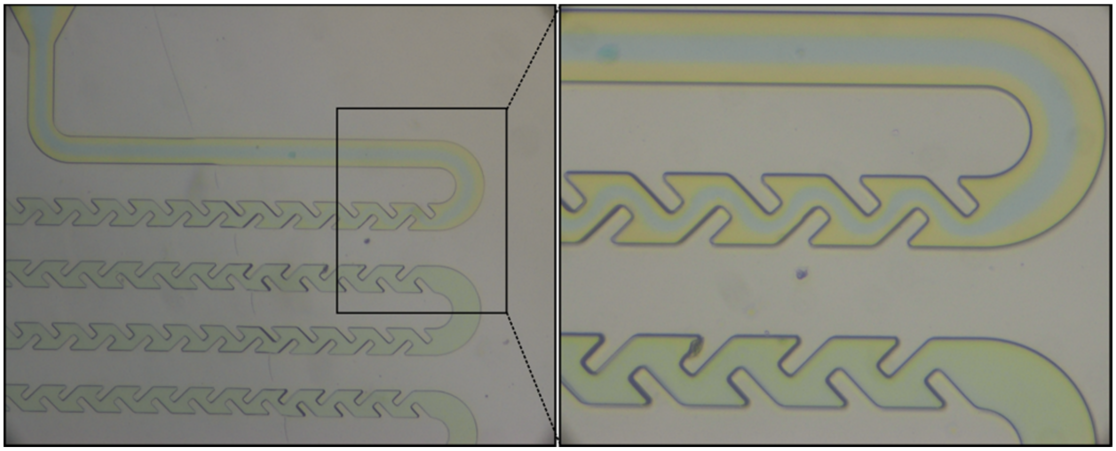

Supplementary Figure 1 Microfluidics device mixing efficiency. Yellow and blue food coloring solutions are introduced to inlets 1 and 2, respectively. After approximately one tenth of the mixing channel length, the solutions are no longer phase separated and the solution color is green.

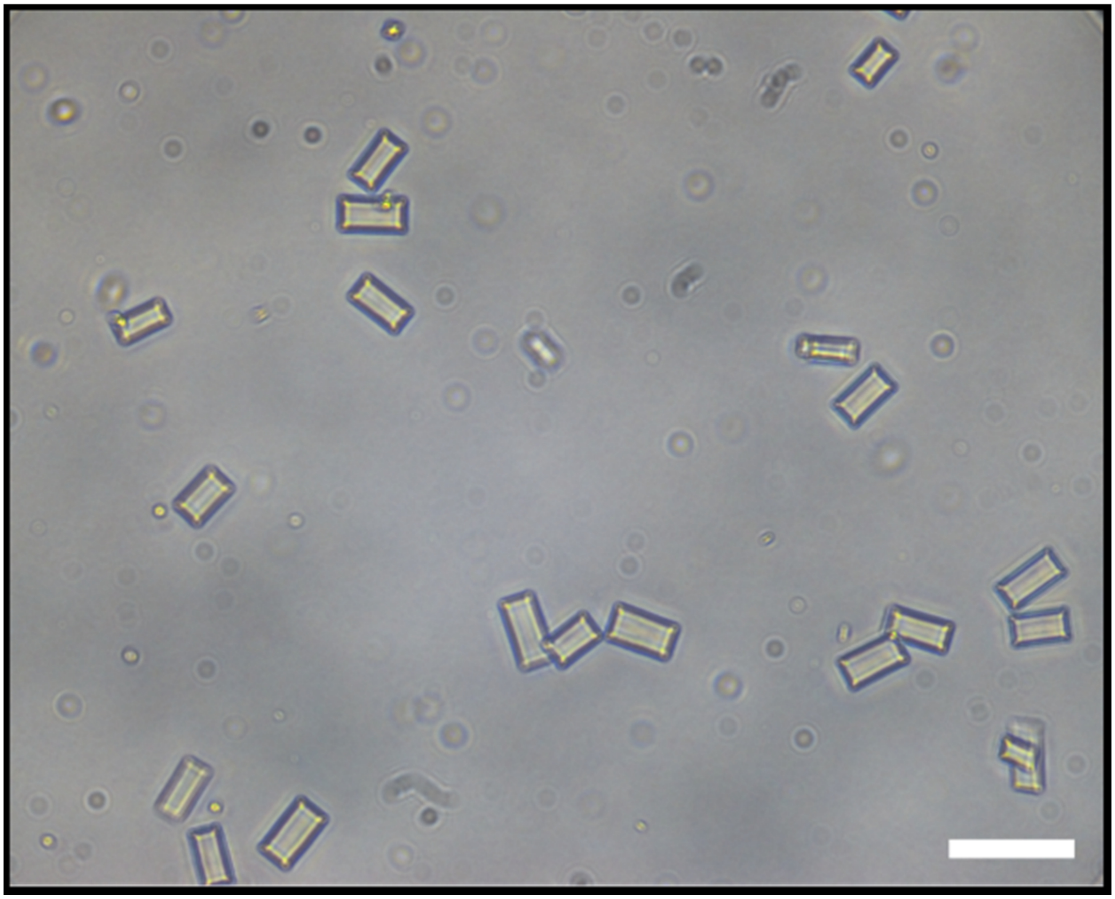

Supplementary Figure 2 FF at concentration of 8 mg/ml dissolved in 100% DMSO. The molecules assemble into plate-like structures. Scale bar is 50 µm.

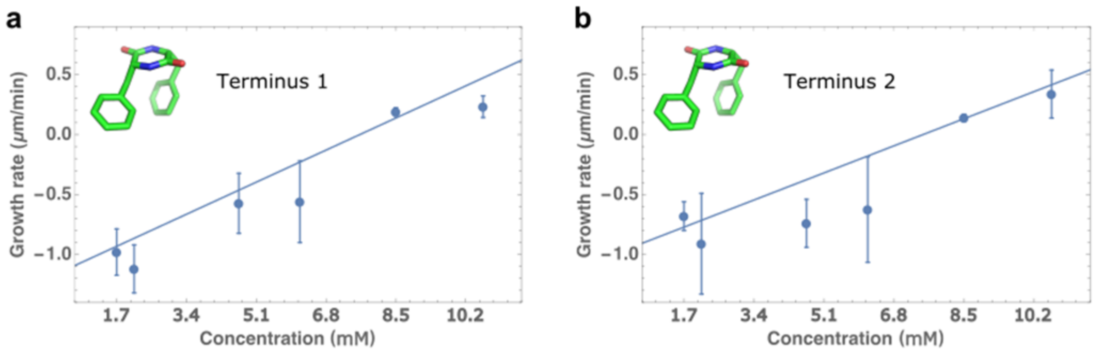

Supplementary Figure 3 CycloFF growth rates for each elongating terminus separately. To differentiate between the two termini, the terminus facing the flow origin was denoted as Terminus 1 and the terminus pointing towards the flow direction was denoted Terminus 2.

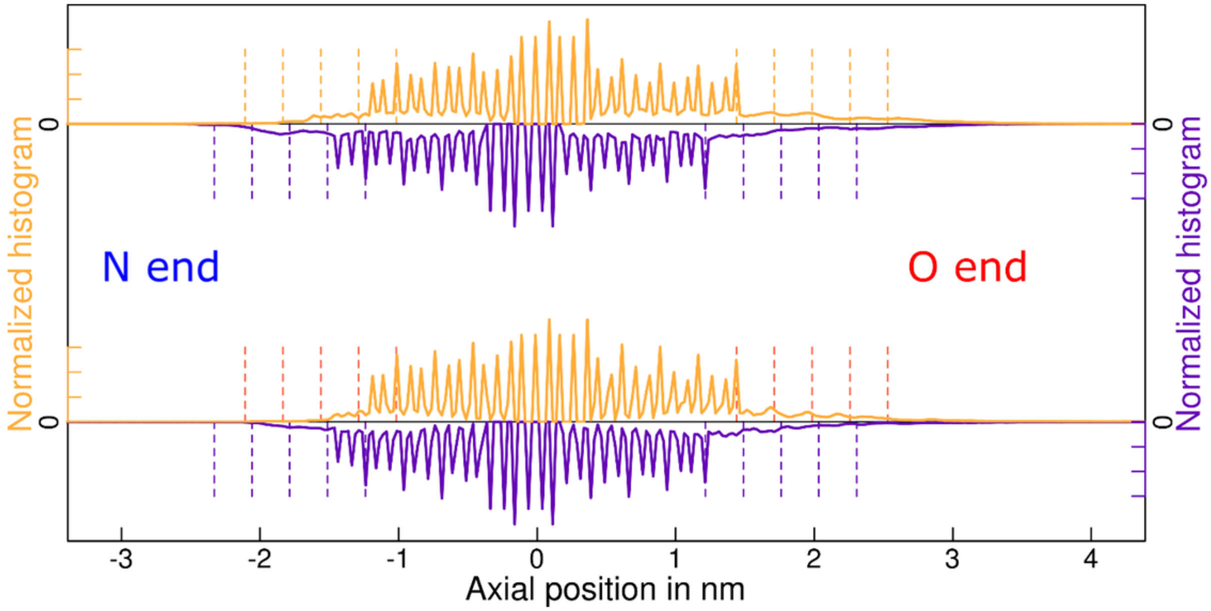

Supplementary Figure 4 Axial density profiles of carboxyl (left y-axes) and amino (right y-axes) groups. The top and bottom halves are for unrestrained and restrained cases, respectively. Vertical dashed lines indicate the most prominent peak positions expected for a systematic continuation of the crystal.

Supplementary Discussion

Microfluidic Platform

In order to ensure a monomeric nature of the solution phase of the peptide, samples were heated to 90°C for several minutes and then mixed thoroughly through vortex treatment. The monomeric solution was injected into the microfluidic device at a low flow rate of up to 4 μl/h with the aim of decreasing the effect of shear stress upon the nanotubes, and to ensure proper mixing within the device. At this slow flow rate, building blocks diffusion through solution is the limiting growth factor, while elongation at faster flow rates is significantly affected by surface effects.

The two flow rates of solutions into the microfluidic channel can be fine-tuned, thus providing instantaneous and direct control over the confined environment within the device. The nanotubes were imaged using light microscopy throughout the process of elongation or shortening. The length of each nanotube termini was analyzed as a function of time and the effective concentration in the microfluidic channel.

Molecular Simulations

We represent the system as a narrow cylinder with length 188 nm and radius 3.7 nm (see Fig. 4A in main text). The cylinder is periodic along the axis and uses a soft wall elsewhere (harmonic restraining force with force constant of 0.2 kcal/(mol·Å^2) acting on atoms). Its axis is aligned with the asymmetric axis of five layers of a performed crystallographic assembly of FF with 70 molecules per layer. The central layer and all molecules outside of the cylinder volume are frozen. These molecules serve to provide a template and reduce boundary artifacts. In addition, there are 70 molecule in the soluble phase (~14mM), which initially are dispersed randomly. Solvent is represented as a continuum (see below). The FF crystal is peculiar in that the packing requires the ϕ-angle of the second phenylalanine residue to be positive. This is an unlikely state in solution, and the energetic balance between positive and negative ϕ-angles as well as the barrier separating them are often described poorly in biomolecular force fields. We thus perform two sets of simulations, the first on an unperturbed model and the second in the presence of conformational restraints on FF. Specifically, the central 3 backbone torsions (ψ, ω, and ϕ) and all four χ-angles were restrained to the crystal conformation with force constants of 0.02 kcal//(mol·deg^2).

The molecular simulations are performed in a torsion angle and rigid-body space and are made up of both molecular dynamics (MD) and Monte Carlo (MC) steps alternating in intervals of 20000 steps. Such a mixed sampler has been used for an unrelated sampling problem before[5] and its primary benefit is the access to different length scales in dilute systems. Incremental and highly correlated changes are provided by the MD engine[4] whereas jumps in individual or few selected degrees of freedom are made available by specialized MC moves. Here, we use the recommended integrator for MD and an MC move set featuring the following move types: 4% global rigid-body moves (coupled translation and rotation), 6% stepwise rigid-body moves (0.4Å, 2°), 5.4% random χ-angle moves, 21.6% stepwise χ-angle moves (5°), 12.6% random ϕ/ψ-angle moves, and 50.4% stepwise ϕ/ψ-angle moves (5°)[3]. The values in parentheses are maximum step sizes taken from a uniform distribution. The random or global moves set the degrees of freedom to randomly selected values from the entire available space. Due

to the size of the system, random rigid-body moves are critical for equilibrating the soluble phase quickly. The MD steps use a time step of 5fs with mass redistribution on the terminal amino groups for improved stability[4]. Constant temperature is maintained by an asynchronous Andersen thermostat with a coupling time of 2ps. Integrator error means that the ensembles provided by MD and MC are not identical, which will be manifest in details of the equilibrium statistics. This is a reasonable tradeoff here due to the systematic model inaccuracy likely outweighing this problem and due to the primary interest in a qualitative observation (growth asymmetry).

Molecular simulations were performed using the ABSINTH implicit solvent model[2], which is developed for the selected degrees of freedom. The crystal structure of FF[1] (see Fig. 3 in the main text) is particular in that it contains water-filled, tubular channels lined by a helical network of salt bridges formed by peptide termini. This involves a very delicate balance of peptide-peptide *vs*. peptide-water interactions, which challenges any classical description. We applied three simple modifications to the published ABSINTH model: 1) the Lennard-Jones size parameters for aromatic carbon and carboxylate oxygen atoms were increased by 0.3 Å each; 2) the reference free energies of solvation corresponding to the model compounds representing the charge peptide termini were set to -91.5 and -92.3 kcal/mol for amino and carboxylate groups, respectively; 3) partial charges and required bonded parameters were taken from the CHARMM22 force field (rather than OPLS-AA/L). Changes 1) and 2) were implemented as a result of test simulations on the system monitoring stability of the crystallographic assembly. Their usefulness in other contexts is under investigation. Change 3) is not specifically linked to the application. All interatomic interactions are truncated at 12 Å. Due to the use of an implicit solvent model with inhomogeneous dielectric, standard treatments of long-range electrostatics such as the particle-mesh Ewald method[6] are not available. Instead, we switch to calculating the monopole-monopole interactions between amino and carboxyl groups at reduced resolution when they are safely beyond the cutoff. Cutoff noise contributes the most to the integrator error, which is manifest as a temperature mismatch of ~4 °C.

All simulations were run with version 3b of CAMPARI (http://campari.sourceforge.net) on the supercomputer Piz Dora (a Cray XC40) at CSCS compiled using Cray compilers. CAMPARI is publicly available free of charge. The latest beta version (3b) and required input files replicating the calculations exactly are available upon request from the authors (campari.software@gmail.com). The number of atoms is 18060. Individual and independent simulations ran for 1.38×10^7 steps at 4 different target temperatures (22, 32, 42, and 52 °C) both with and without the aforementioned conformational restraints. The initial conformations for the 70 soluble molecules were created randomly and independently for every run.

We computed the oligomer state of all molecules by monitoring interatomic contacts using a threshold of 4 Å. Molecules being part of assemblies having less than 10 molecules were considered soluble (Fig. 4B). Under all conditions investigated, at least 85% of these small oligomers were monomers. To characterize the asymmetry of the system, we calculated the number of binding and unbinding events at either interface (Fig. 4C). To do so, the oligomeric status of every molecule was monitored over time. Whenever a molecule transitioned from a small (≤10) to large (>10) oligomer in a region extending up to 40 Å into solution from the central, ordered layer, a binding event was

recorded. The analogous procedure was followed for unbinding. This is robust since the populations of oligomers with intermediate sizes (between 10 and 300) was zero throughout. Finally, we computed axial density profiles for the simulations at 22 °C (Fig. 4D). For these, the centers of mass of the carboxyl and amino groups were computed and binned along the cylinder axis using a bin size of 0.25 Å. These data are collected only over the latter half of each simulation. Extended Data Fig. 1 shows the axial density profiles of carboxyl (left y-axes) and amino (right y-axes) groups. All Cartoon insets to Fig. 4 were rendered with VMD[7].

Supplementary Methods

Materials

Peptides were purchase from Bachem, Switzerland (purity ≥97%). Fresh stock solutions were prepared by dissolving FF in water at concentrations of 1, 0.76 and 0.5 mg/ml and cycloFF in Dimethyl sulfoxide (DMSO) at a concentrations of 0.5 and 4 mg/ml. Preformed structures were assembled by dissolving FF in water at concentration of 2 mg/ml and cycloFF in DMSO at concentration of 10 mg/ml and heating to 90 °C. Structures were visible when samples were cooled down to room temperature.

Microfluidic Device

Microfluidic device was designed using CleWin software (CleWin Layout Editor Version 4.1.2.0, Micro and Nano Fabrication and Characterization Facility, Tel Aviv University) (Extended Data Video 1) and fabricated with poly(dimethylsiloxane) (PDMS), using SU8 on silicon masters and standard soft lithography techniques. Inlets and outlets were punched and PDMS was then plasma bonded to glass slides to create sealed devices. The main channel of the device, which includes the pillars, is 1 mm wide and 8 mm long. Pillars dimensions are 50x25 μm. The width mixing channel varies berween 30 – 100 μm. The height of the channel is 50 μm.

Microfluidic Experiments

Preformed crystalline structures were inserted into the device. Then, a flow of solutions of known concentrations was injected at a rate of 4 μl/h using Cetoni GmbH neMESYS Syringe Pumps (Korbussen, Germany) and glass HAMILTON syringes, 1725 TLL of 250 μl. This process was examined under an OPTIKA XDS-2 Trinocular Inverted microscope, and images were captured at different time points. See Supplementary Text for further information.

Image Analysis

Captured images were analyzed using ImageJ 1.45S. The length of the tubes was measured at all time points. Growth and shortening rates were calculated by including only those tubes which had both ends visible in the image frame for the entire process. The length of each end was measured for 5 times and averaged for each time point.

Crystal Structure

Crystal structures 2x2x2 packing images were illustrated using Mercury 3.3. Crystal monomer images were represented using PyMol 1.3.

Molecular Simulations

All details regarding the setup and analysis of molecular simulations are provided as SI. Images were generated using VMD 1.9.2 and R 2.14.

Supplementary References

1. Görbitz, C. H. The structure of nanotubes formed by diphenylalanine, the core recognition motif of Alzheimer's beta-amyloid polypeptide. *Chem. Commun. (Camb).* 2332–2334 (2006).
2. Vitalis, A. & Pappu, R. V. ABSINTH: a new continuum solvation model for simulations of polypeptides in aqueous solutions. *J. Comput. Chem.* **30,** 673–699 (2009).
3. Vitalis, A. & Pappu, R. V. Methods for Monte Carlo simulations of biomacromolecules. *Annu. Rep. Comput. Chem.* **5,** 49–76 (2009).
4. Vitalis, A. & Pappu, R. V. A simple molecular mechanics integrator in mixed rigid body and dihedral angle space. *J. Chem. Phys.* **141,** 034105 (2014).
5. Vitalis, A. & Amedeo C. Equilibrium sampling approach to the interpretation of electron density maps. *Structure* **22,** 156-167 (2014).
6. Essmann, U. et al. A smooth particle mesh Ewald method. *J. Chem. Phys.* **103,** 8577-8593 (1995).
7. Humphrey, W., Andrew D., & Klaus S. VMD: visual molecular dynamics. *J. Mol. Graph.* **14,** 33-38 (1996).
